# When Families Choose Sons: Parental Gender Norms and Girls’ Education in Ghana

**DOI:** 10.3390/populations1040025

**Published:** 2025-11-28

**Authors:** Portia Buernarkie Nartey, Proscovia Nabunya, Peace Mamle Tetteh, Fred M. Ssewamala

**Affiliations:** 1 International Center for Child Health and Development (ICHAD), Brown School, Washington University in St. Louis, St. Louis, MO 63130, USA; 2 Department of Sociology, University of Ghana, Accra P.O. Box LG 65, Ghana

**Keywords:** gender norms, female education, educational disparities, parental attitudes, Ghana

## Abstract

Despite global progress toward gender parity in education, Ghanaian girls continue to face systemic barriers rooted in entrenched parental gender norms. This paper explores how parental gender norm beliefs and attitudes perpetuate disparities among school-aged, particularly disadvantaging girls in access to and retention in education. Using a desk review methodology, we analyzed peer-reviewed social science and development literature, legal documents, and international reports from organizations such as the United Nations and the World Bank to explore the structural and cultural dynamics affecting girls’ education in Ghana. Anchored in Social Impact Theory, Parental Ethnotheories, and Expectation States Theory, the study provides a multi-theoretical lens to understand how gender norms, cultural expectations, and parental beliefs converge to influence educational outcomes for girls. Analysis of sociocultural norms, economic trade-offs, and safety concerns reveals how parents—often guided by love and pragmatism—prioritize sons’ education while withdrawing daughters for caregiving, early marriages, or income-generating labor. The study highlights three critical dimensions: (1) the economic reasoning behind gendered investments in children’s schooling, (2) sociocultural gender norms limiting girls’ retention in school, and (3) the transformative potential of educated women as community leaders challenging these patterns. Evidence shows that educating girls yields broad benefits, from improved health outcomes to economic growth, yet systemic inequities remain. Findings underscore the need for interventions to move beyond school access to address the familial and cultural ecosystems shaping parental decisions. By disrupting entrenched gender norms, Ghana can advance SDGs 4 and 5 and promote long-term societal change.

## 1. Introduction

A target of the Sustainable Development Goal (SDG) 4 is eliminating gender disparities in education by 2030. Over the past 25 years, girls’ access to school has advanced generationally [1]. While there has been significant progress, it is widely acknowledged that education outcomes for girls in low-income countries continue to fall behind those of boys [2]. These gender disparities are caused by deeply engrained discriminatory gender norms that continue to lower the value of girls’ education, impede access, and negate the advantages of girls’ and young women’s access to education in sub-Saharan Africa (SSA) [3]. This is evident in Ghana, where parental gender norm beliefs and attitudes often prioritize boys’ schooling, reflecting broader sociocultural and economic inequities [4].

While the detrimental effects of gender norms on girls’ education in Ghana are well-documented [2,5], existing literature often examines these norms in isolation, such as focusing either on economic, sociocultural, or safety-related barriers. A critical gap remains in the theoretically integrated synthesis of how multiple, interconnected parental gender norm belief systems operate simultaneously to influence educational outcomes for girls. Furthermore, many studies are empirical and localized, leaving a need for a comprehensive, national-level analysis that explicitly connects parental decision-making to established social psychological theories. This desk review addresses this gap by applying a multi-theoretical framework (Social Impact Theory, Parental Ethnotheories, and Expectation States Theory) to synthesize existing evidence and provide a cohesive model of how parental norms function at the nexus of culture, economy, and safety. For policymakers and practitioners, this review moves beyond cataloging barriers to explain the underlying cognitive and social processes in parental decision-making, thereby informing the design of more nuanced, theory-driven interventions that can disrupt these deeply ingrained norms. This review also aims to inform educators, policymakers, development practitioners, and researchers working in education and gender equity about the role of household-level gender norms in shaping girls’ access to and retention in school. By highlighting key patterns and challenges, this article seeks to support the design of more culturally responsive and gender-transformative interventions [6] to improve the educational outcomes for adolescent girls in Ghana.

This review is therefore guided by the following research questions:

1. How do Social Impact Theory, Parental Ethnotheories, and Expectation States Theory collectively explain the mechanisms through which parental gender norms influence girls’ access to, retention in, and achievement within the Ghanaian education system?
2. What are the predominant sociocultural, economic, and safety-related manifestations of these parental gender norms?

## 2. Theoretical Framework

We used Social Impact Theory (SIT), Parental Ethnotheories, and Expectation States Theories as frameworks for understanding how parental gender norms influence adolescent girls’ schooling and education. Latané (1981) defines social impact as any influence on individual feeling, thoughts, or behavior that is exerted by the real, implied, or imagined presence or actions of others [7]. Social impact has been used to demonstrate the influence of groups on an individual, such as followers influencing a leader [8]. Social Impact Theory (SIT) has also been used to determine college students’ assessment of the drinking behavior of their peers when alone and when surrounded by members of their group [9]. The most fundamental understanding of SIT contends that the capacity of a social context to affect an individual depends on its strength (that is, status, age, prior relationship with, or authority over the target), immediacy (closeness in time or distance), and number of sources of influence (number of people) [7]. Therefore, in a social environment, the more powerful, immediate, and numerous the sources of influence are, the more impact the targeted individual will experience [10]. In this case, the gender norms and values of a parent or family relative are more likely to influence an adolescent girl than the gender belief of one who is not closely related. Thus, parental beliefs and actions about the value of a girl’s education have a strong influence on how adolescent girls view and participate in education.

Parental ethnotheories are parents’ beliefs and understanding about gender norms and child education shaped by their cultural upbringing and societal context [11]. How parents conceptualize gender norms and child education is a reflection of their own socialization, which in turn influences parental actions and decisions about children and their development in systematic ways [11]. Moreover, parental ethnotheories shape parents’ beliefs about gender roles within the family and society. For example, in some cultures, girls are expected to prioritize their household responsibilities over their academic career aspirations, while boys are expected to prioritize their academics because gender norms position them as the primary breadwinners [12]. These beliefs can influence parents’ decisions about how they allocate resources and opportunities to their children’s education based on their gender. Indeed, parents prioritize boys’ education over girls’ based on the perceived higher rates of return to boys’ education than girls’ (RORE) [2].

While Social Impact Theory and Parental Ethnotheories help explain how broader community norms and cultural beliefs shape parental decision-making, Expectation States Theory [13] adds a critical dimension by illuminating how those norms translate into implicit hierarchies of competence and value. According to this theory, individuals form performance expectations—often unconscious—based on socially constructed characteristics such as gender. These expectations significantly influence behavior, resource allocation, and opportunities for success [13]. In the context of Ghana, many parents hold the belief—rooted in entrenched gender norms—that boys are more academically capable or more likely to benefit from formal education. As a result, boys often receive disproportionate investments in their education, such as school fees, study time, or academic encouragement [2,14]. Conversely, girls, who are often socialized to embrace roles as future wives and caregivers [12], are perceived as less likely to excel academically. This perception creates a self-fulfilling cycle: low expectations lead to fewer investments, which then result in fewer opportunities to succeed—thus reinforcing gender norm beliefs.

Expectation States Theory emphasizes that people tend to perform in line with the expectations others hold for them [13]. When high expectations are placed on an individual, parents and communities are more likely to implement structures and provide resources to support that individual’s success [2]. On the other hand, when expectations are low or nonexistent—as is often the case for girls under restrictive gender norms—parents are less likely to invest in the child’s education, believing it to be a poor or unnecessary return on resources [2,15]. Moreover, even when girls are granted access to education, parental expectations often guide them toward traditionally gendered subjects and careers (e.g., caregiving, homemaking), while boys are encouraged to pursue prestigious and higher-paying fields such as engineering or medicine [16–18]. These educational and career pathways mirror underlying assumptions about competence and worth, perpetuating gender inequality and limiting girls’ potential. Thus, Expectation States Theory underscores the importance of confronting and reshaping parental expectations as a pathway to promoting equitable access to education and achievement for girls in Ghana.

**Figure 1.**
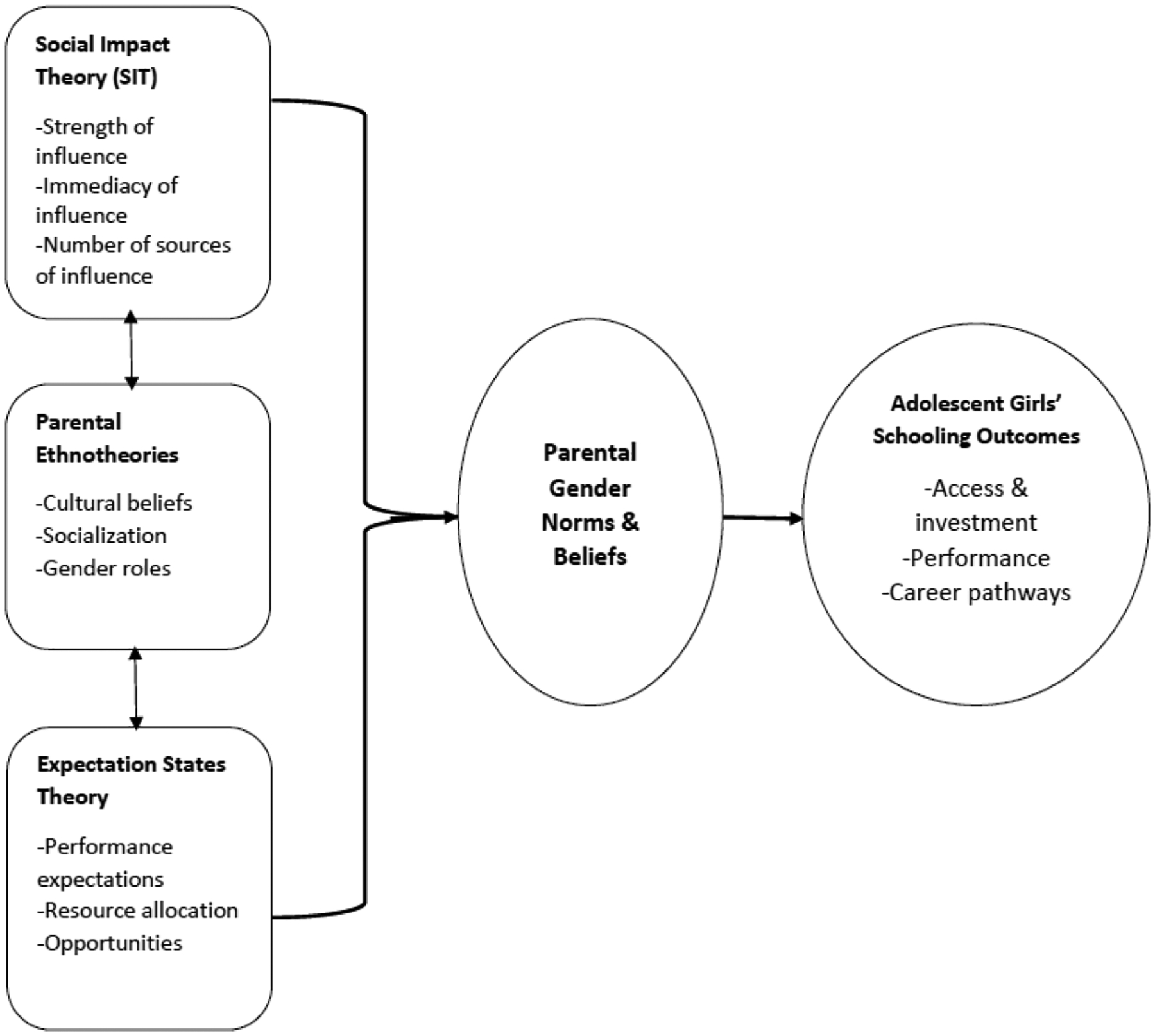
A Conceptual Framework Integrating Three Theories to Explain the Impact of Parental Gender Norms on Girls’ Education.

Together, these frameworks form a sequential and reinforcing chain that explains how parental gender norms shape girls’ education in Ghana. SIT highlights how families absorb external pressures from communities and cultural expectations that often privilege sons. Parental Ethnotheories show how these pressures become internalized as parental beliefs about the relative value of educating boys versus girls. Expectation States Theory then clarifies how such beliefs are enacted—shaping expectations, investments, and schooling outcomes. Collectively, the three theories connect broader social forces, parental worldviews, and concrete educational decisions, illuminating why families may prioritize sons’ schooling over daughters. See Figure 1 below for a visual illustration of the integration of the three theories.

## 3. Methods

This study employed a narrative review methodology through a desk review to identify and synthesize existing literature on parental gender norms and female education in Ghana. Sources were drawn from peer-reviewed social science and development journals such as Gender, Place and Culture, the Journal of Child and Family Studies, and the Journal of Marriage and Family. In addition, relevant legal frameworks, policy documents and reports from international organizations, including United Nations agencies and the World Bank, were explored to supplement the review with authoritative data on the state of girls’ education in Ghana. The search strategy combined keywords related to three concepts: (1) Gender norms (“gender norm” OR “parental attitude” OR “sociocultural norm” OR “gender role” OR “parental expectation*”); (2) Education (“girl* education” OR “female education” OR “educational disparit*” OR “school enrollment” OR “school dropout” OR “educational access”); and (3) Geography (“Ghana”). The search was limited to literature published in English between 2000 and 2024 to focus on more recent publications. Studies were included if they: (1) explicitly examined gender norms, parental attitudes, or expectations as a central theme; (2) focused on educational access, retention, achievement, or decision-making for school-aged children; and (3) presented findings specific to the Ghanaian context or included Ghana in a multi-country analysis with disaggregated insights. Studies were excluded if they focused solely on general poverty or economic barriers without a specific gender norms lens. Findings were synthesized as guided by the three primary theoretical frameworks: SIT, Parental Ethnotheories, and Expectation States Theory.

## 4. Parental Gender Norms and Barriers to Girls’ Education in Ghana

### 4.1. Sociocultural Barriers

Gender norms—the socially constructed expectations that define appropriate roles, behaviors, and responsibilities for males and females—operate at multiple levels of society [19]. They shape how families and communities perceive the duties and capacities of boys and girls, influence what is taught in schools and how it is taught [20], determine how men and women are portrayed in the media [21]. and even structure religious teachings about authority and submission [22,23]. Because teachers, community leaders, and parents are all products of these social environments, they internalize and reproduce these gendered expectations, reinforcing them through everyday interactions and institutional practices [22,23].

In Ghana, these gender norms are deeply embedded within sociocultural and economic systems and profoundly shape parental beliefs about children’s education. Parental gender norms—the deeply held beliefs about appropriate roles for sons and daughters—create significant sociocultural barriers to girls’ education. They manifest in how parents allocate household responsibilities, with daughters typically burdened with domestic chores like cooking, cleaning, and childcare while sons are exempt [4,24]. Parents actively socialize their children into these gendered roles, training girls for wifehood and motherhood while encouraging boys’ academic and career aspirations [14]. This differential treatment stems from gender norm beliefs that a woman’s primary value lies in her domestic abilities rather than formal education [25]. The allocation of domestic chores to daughters is a direct manifestation of Parental Ethnotheories that equate a girl’s value with her domestic capabilities [11].

These norms influence family perceptions of children’s duties, shape school curricula and teaching methods [20,26], and are reinforced through media portrayals and religious teachings that prescribe distinct roles for men and women [21]. Even educators, who are themselves products of these communities, often internalize and perpetuate these norms through their classroom practices and attitudes [22,23]. Parents play the most critical role in gender socialization, actively shaping their children’s understanding of gender roles through both direct instruction and modeled behavior [27,28]. From infancy, children observe and internalize gendered divisions of labor—seeing mothers primarily as caregivers while fathers engage in leisure activities [29]—and by adolescence, these norms are fully entrenched, creating significant disparities in educational access and achievement [23]. In Ghana, these sociocultural norms take concrete form through practices like child marriage, particularly in northern regions where bride price systems (involving payments of livestock or money to the bride’s family) incentivize early marriage [30,31]. While gendered barriers to girls’ education exist nationwide, their intensity and expression vary across regions. In parts of northern Ghana, sociocultural expectations around early marriage and domestic responsibility tend to be more deeply entrenched, shaped by intersecting economic hardship and traditional value systems [12,30,31]. In southern and urban areas, these barriers often manifest differently—through subtle but persistent expectations about female domestic roles and family care responsibilities [4]. Many parents prioritize training girls for domestic roles and future wifehood over formal education [12], a preference further reinforced by religious and traditional beliefs that position women primarily as wives and mothers [25,32]. These beliefs continue to shape parental choices because, as SIT suggests, the ‘strength’ and ‘immediacy’ of influential figures—grandparents, religious leaders, and community elders—sustain these norms and make resistance difficult [7].

### 4.2. Economic Factors

Parental gender norms fundamentally shape economic decisions about children’s education, with sons consistently prioritized due to perceived higher returns on investment [2,33]. Ghanaian parents calculate the opportunity costs of educating daughters differently than sons, considering both immediate labor needs and long-term benefits [2,4]. The calculation of higher returns on investment for sons’ education is not merely a financial decision but is underpinned by the performance expectations outlined in Expectation States Theory [13]. Parents, acting on the socially constructed belief that boys are more competent and will be more successful, invest resources in a way that becomes a self-fulfilling prophecy, thereby perpetuating the economic disparity. Across SSA, such gendered calculations contribute to a persistent educational gap, as discriminatory gender norms and the unequal burden of domestic responsibilities placed on girls raise the opportunity costs of their schooling compared to boys [2]. In low-income households, adolescent girls are routinely expected to take on extensive caregiving and domestic duties to enable their mothers to engage in paid work outside the home [23]. While this arrangement may provide short-term economic benefits for families, it systematically devalues girls’ education by positioning their labor as more immediately valuable than their schooling [4,20]. The economic barriers to girls’ education are multifaceted: the time poverty created by endless domestic chores including cooking, cleaning, and especially time-consuming tasks like water collection directly reduces the time available for studying and school attendance [24]. World Bank research has quantified this impact, showing that reducing water-fetching time by just 50% could increase girls’ school enrollment rates by 2.4 percentage points [34]. When families face financial constraints, parents overwhelmingly prioritize boys’ education due to the perception that sons will provide greater long-term economic returns, as they typically remain in the household as adults, while daughters’ labor and potential earnings are lost upon marriage [2,35,36]. This economic calculus leads to heartbreaking choices for girls. For example, in northern Ghana, girls as young as 12 years are made to engage in hazardous work to earn income to financially contribute to the household, and support their family and male siblings’ education [37]. These economic dynamics also vary by region and class; while northern households often face acute poverty that reinforces early marriage and child labor, lower-income families in urban centers in more urban southern households confront different trade-offs—such as balancing school attendance with informal market work [12]. The consequences of these economic barriers are visible in national statistics: only 11.7% of Ghanaian girls complete secondary school compared to 18% of boys [35], while across SSA, female literacy rates (75.4%) continue to lag behind male rates (79.2%) [38].

### 4.3. Safety and Access Barriers

Beyond sociocultural and economic barriers, girls in Ghana face significant physical and social risks that further limit their educational opportunities. The simple act of getting to school can be dangerous, particularly in rural areas where children must traverse long distances on foot, often exposing them to risks of kidnapping or sexual violence [5,36]. These dangers are compounded by a near-total lack of transportation infrastructure in many communities. Even when girls do reach school, the environment itself may not be safe, with persistent risks of sexual exploitation by both teachers and male peers [18]. For many parents, fears that school attendance might lead to premarital pregnancy—a culturally stigmatized outcome—serve as a powerful deterrent to enrolling their daughters in school [39]. These fears are exacerbated by the reality that many schools lack proper safeguards, with some teachers themselves perpetuating sexual abuse [22].

Parents’ decisions to withdraw adolescent girls from school reflect not just practical safety concerns but deeply ingrained gender norm beliefs about female sexuality and respectability [12]. The gendered nature of these safety calculations is evident in how differently parents treat sons and daughters—while boys are encouraged to travel independently to school, girls are restricted due to perceptions of vulnerability [4,5]. These protective measures, though well-intentioned, ultimately limit girls’ educational opportunities. Parental fears about safety and premarital pregnancy are filtered through the lens of Parental Ethnotheories about female purity and the role of women. Expectation States Theory clarifies that lower expectations for girls’ future public achievement make the risk of sending them to school seem less worthwhile [13]. Meanwhile, SIT illustrates how community gossip and stigma (‘number of sources’) powerfully enforce these protective-but-restrictive decisions [7]. Even when Ghana’s Free Compulsory Education policy provides access, parental gender norms about safety may prevent many girls from benefiting from such a transformative policy [40]. The cumulative impact of these barriers is starkly evident in regional education statistics: UNESCO estimates that four million girls across SSA may never enroll in school at all, compared to just two million boys [41], while in 80% of SSA countries, female lower secondary school completion rates remain below 60% [38]. These numbers represent not just educational disparities but the systematic exclusion of girls from opportunities to improve their lives and transform their communities.

## 5. The Transformative Benefits of Girls’ Education

While the advantages of girls’ education are well documented, their realization depends on transforming the parental gender norms that determine whether girls are allowed and supported to stay in school. Drawing on SIT, Parental Ethnotheories, and Expectation States Theory, this section analyzes how shifts in parents’ beliefs about girls’ roles and capacities activate the health, economic, and sociocultural benefits of education. In this sense, education is not only an outcome of empowerment but also a mechanism that reshapes the very norms constraining girls’ opportunities in Ghana.

### 5.1. Health and Reproductive Outcomes

Education serves as one of the most effective tools for improving health outcomes among girls and young women. In SSA, adolescent girls bear a disproportionate share of the HIV burden [42,43]. Recent data from 2023 indicate that girls (ages 15–19) accounted for roughly 84% of new HIV infections among adolescents in the region, with around 750,000 adolescent girls currently living with the virus [42,44]. In this region, where adolescent girls face disproportionately high HIV infection rates exceeding 30% in some countries [45], education provides critical protection through multiple pathways [46–48]. However, these protective effects become sustainable only when parents abandon norms that equate female virtue with early marriage and domestic obedience. When parental ethnotheories shift to value girls’ education as a form of protection and empowerment rather than risk, families actively support continued schooling, reinforcing positive health outcomes. School attendance delays sexual debut and first marriage while equipping girls with the knowledge and confidence to negotiate safer sexual practices [49]. Educated females demonstrate higher rates of condom use and reduced engagement in transactional sexual relationships with older male partners, who are more likely to be HIV-positive [45,50,51]. The protective effect of education is quantifiable. For instance, in Uganda, HIV infection rates declined most sharply among young women with secondary education [45], while UNAIDS identifies female education as a cornerstone of HIV prevention strategies [52].

Beyond HIV risk reduction, maternal education significantly improves reproductive health outcomes. Educated women marry later, have fewer children, and utilize family planning services more effectively [53,54]. Their children benefit directly, infants born to mothers with secondary education face significantly lower mortality risks and receive better antenatal care compared to those born to uneducated mothers [53]. These health advantages create a virtuous cycle, as healthier families can invest more resources in education for the next generation. Access to education empowers females with the knowledge and skills needed to make informed decisions about their health and that of their families [55].

### 5.2. Economic Empowerment and Labor Participation

Transformative economic benefits emerge when parents begin to view daughters as capable economic contributors rather than dependents. According to Expectation States Theory, when parents revise performance expectations for girls’ future success, they make more equitable investments in their daughters’ education, catalyzing household-level economic mobility [13]. The economic returns on female education transform both individual livelihoods and national economies. Each additional year of secondary schooling increases a girl’s earning potential as an adult by 15–25%, with ripple effects across households and communities [26,56]. Educated women participate more actively in the formal labor market, with studies showing that secondary school completion correlates with 40% higher lifetime earnings [53,57,58]. This economic mobility enables women to invest approximately three times more in their children’s education compared to less-educated peers [56], breaking intergenerational poverty cycles. At the macroeconomic level, the impact is equally profound, cross-national research demonstrates that increasing average educational attainment by one year can boost annual GDP growth by 3.7% in developing countries [53,59,60]. In line with this, Ghana’s implementation of the Free Senior High School policy has contributed to increased female secondary school enrollment, positioning the country to potentially realize similar economic gains [61]. When girls stay in school, they gain access to skilled employment opportunities in emerging sectors like technology and finance, while developing the entrepreneurial capabilities needed to thrive in small and medium enterprises—a critical driver of a nation’s economic diversification [53,56,62].

### 5.3. Societal Transformation and Intergenerational Impacts

The societal benefits of educating Ghanaian girls extend far beyond individual achievement, reshaping gender norms and community development trajectories. Educated women exhibit dramatically increased participation in household decision-making and local governance, applying their literacy skills to advocate for community needs [17,45,63,64]. As recognized at the 1995 Beijing Conference, educated mothers are more likely to champion girls’ education, creating a generational multiplier effect [53]. Their families demonstrate better health literacy, with 30% higher nutrition standards and more equitable resource allocation between sons and daughters [54]. Educated women are also more likely to engage in community health initiatives, support preventive practices, and advocate for improved healthcare services [55]. At the national level, educated women drive democratic strengthening through increased civic engagement and political representation [55,57,58]. Research across 19 developing countries confirms that female education correlates strongly with more stable institutions and accountable governance [53]. Perhaps most significantly, the children of educated women—particularly daughters—are three times more likely to complete secondary education themselves [56], ensuring these transformative benefits compound across generations. Ghana’s progress toward SDG 4—reflected in a secondary school completion rate of 74.1% and a literacy rate of 93.5% among those aged 15–24 [65]—therefore represents not just educational advancement, but a fundamental restructuring of social and economic systems to empower women as agents of sustainable development.

Education catalyzes profound societal shifts that extend beyond individual beneficiaries to reshape families and communities. In Ghana, educated women demonstrate a greater likelihood of investing in their children’s education compared to their uneducated peers [53,56], creating a virtuous cycle that gradually erodes traditional preferences for sons’ schooling. These intergenerational effects manifest most visibly in health outcomes: children born to mothers with secondary education experience lower mortality rates and benefit from improved nutrition standards [53,54,66]. At the community level, educated women correlate with broader norm shifts, including delayed marriage ages and increased school enrollment for girls [55]. Critically, educated mothers actively challenge the gendered division of domestic labor [67]. This quiet revolution in homes and communities lays the groundwork for systemic change, as each educated generation models new possibilities for the next [55]. This broader societal change reflects the cumulative impact of many families re-socializing daughters in ways that challenge traditional role expectations. As SIT suggests, when influential community members—parents, elders, educators—model gender-equitable behaviors, the perceived social costs of supporting girls’ education decline, accelerating norm change [7].

### 5.4. Political Voice and Decision-Making Power

While these societal shifts emerge organically from educated populations, true gender parity requires intentional political engagement. This trajectory reflects the cumulative effects of expanded agency and shifting household power dynamics resulting from parental norm change [68]. This transformation unfolds first within the home, where educated women dismantle generations of gendered decision-making [17,63,64]. An educated woman with secondary education is more likely to control her household’s budget than her unschooled counterpart [68]. Additionally, in educated mothers’ homes, women contribute to the final decision on children’s schooling—a dramatic reversal of the traditional norm where fathers alone decide children’s educational futures [69]. These domestic power shifts ripple outward, as educated women bring their confidence and analytical skills to community spaces. As these women gain confidence, their influence radiates outward into community leadership roles, evidenced by Ghana’s modest but meaningful rise in female Municipal/District Chief Executives (MMDCEs) from 14% (in 2017) to 15% (in 2021) [70]. The culmination of this transformation emerges in national politics. Though women still occupy only 14.5% of parliamentary seats—Ghana’s highest proportion since independence in 1957—each percentage point represents hard-won progress [70]. Notably, female legislators sponsor more bills on education and gender equity than their male peers, with a particular focus on removing the very barriers they overcame [55,70].

What makes this political revolution unique is its grounding in everyday realities. The educated woman who negotiates her daughter’s school fees today becomes the community leader who advocates for free sanitary pads in schools tomorrow, then evolves into the parliamentarian who institutionalizes gender-responsive budgeting. Each step erodes the patriarchal gender norms that once made female voices and impact synonymous with silence. Educated women who exercise political voice often come from families that rejected restrictive gender norms [71]. Their emergence as leaders thus exemplifies the intergenerational payoff of early parental norm shifts that validate girls’ aspirations and public participation.

Overall, the transformative benefits of girls’ education in Ghana cannot be separated from the transformation of the parental and community norms that condition access to it. Through the combined lenses of Parental Ethnotheories, Expectation States Theory, and SIT, this analysis illustrates that education both depends on and reinforces shifts in gendered expectations—creating a feedback loop that gradually dismantles the structural and ideological barriers to gender equity.

## 6. Implications for Policy, Practice and Research

This paper highlights the urgent need for coordinated cross-sectoral efforts to dismantle the deeply rooted gender norms that continue to hinder girls’ education in Ghana. For policymakers, the evidence presents both a challenge and a clear path forward: existing interventions must move beyond expanding access and begin addressing the underlying social and cultural drivers that influence parental decision-making.

While Ghana’s Free Senior High School (FSHS) [72] policy represents a groundbreaking step toward equitable access, it must be strengthened with gender-responsive measures—such as targeted scholarships for girls in under-resourced communities and transportation support to address safety concerns that disproportionately affect female students. These additions could help mitigate some of the structural barriers that keep girls out of school. In addition, health policies targeting the wellbeing of adolescent girls should not exclude parents. Instead, they should intentionally involve parents, recognizing that their beliefs and attitudes strongly influence girls’ health and wellbeing behaviors.

At the community level, entrenched norms that restrict girls’ potential can be strategically transformed into instruments of empowerment. Traditional leaders and local authorities could play a pivotal role by reinterpreting cultural practices to honor and celebrate female academic success, thereby reshaping what is seen as possible and valuable for girls. Parents can also be engaged through existing community gatherings such as Parent-Teacher Association (PTA) meetings, where they are sensitized to the importance of girls’ education and empowered to lead conversations that prioritize and promote educational opportunities for their daughters.

Teachers and school administrators are uniquely positioned to serve as both mirrors of societal norms and agents of change. Professional development programs should be designed to help educators recognize and counteract implicit gender biases—such as favoring boys in classroom interactions or assigning girls stereotypical roles during school activities, including subject/course selections or majors. When replicated across schools, even small pedagogical shifts can collectively reshape how children understand and internalize gender norms from an early age.

Future research should more deliberately involve parents as key stakeholders in interventions focused on girls’ education and gender equity. Actively engaging parents in both the design and implementation of such studies can help shift parental attitudes and beliefs, fostering a deeper appreciation for the value of educating daughters and the long-term costs of discriminatory norms. In sum, a multidimensional strategy that integrates policy reform, community engagement, gender-sensitive pedagogy, and participatory research offers the most promising path toward transforming girls’ education in Ghana—from a privilege granted to some to a right guaranteed for all.

While this review offers a national perspective, meaningful progress requires attention to the diverse realities within Ghana. Regional, socioeconomic, and urban–rural differences create distinct pathways through which gender norms shape parental decision-making and girls’ educational opportunities. For example, girls in rural Ghana encounter more visible constraints from the higher prevalence of child marriage, while urban girls may face different pressures related to safety and social expectations. Likewise, girls from the most economically disadvantaged households—or those with intersecting vulnerabilities such as disability—often require tailored supports beyond universal policy measures. Future interventions and research must therefore avoid a one-size-fits-all approach and prioritize context-specific adaptation, recognizing that transforming gender norms requires reshaping the beliefs and expectations that guide parental choices in each community.

## 7. Strengths and Limitations

A key strength of this article is that it not only highlights the challenges girls face in accessing education but also emphasizes the transformative benefits that emerge when parental gender norms shift—offering a strong foundation for more targeted interventions. Additionally, the article addresses a critical yet often overlooked determinant of female education in Ghana, parental gender norms. While many girls’ education studies focus primarily on poverty, this paper sheds light on how entrenched gender norms, beliefs, and attitudes can significantly hinder girls’ educational opportunities, regardless of available economic resources. Importantly, this study is rigorously guided by three empirical theories—SIT, Parental Ethnotheories, and Expectation States Theory—which collectively provide a strong conceptual foundation for analyzing how parental gender norms, cultural expectations, and beliefs intersect to shape educational outcomes for girls. The use of multiple theoretical lenses enhances the analytical rigor of this study and strengthens its ability to generate nuanced, contextually relevant insights that can inform future research and policy efforts.

However, the study is limited by its reliance on secondary data reports. The absence of primary data means it does not capture firsthand perspectives from parents or adolescent girls on how gender norms shape educational outcomes.

## 8. Conclusions

This study highlights how persistent parental gender norms continue to limit girls’ educational opportunities in Ghana, despite decades of policy reforms. Deep-rooted beliefs about gender roles, shaped by sociocultural traditions, economic pressures, and safety concerns, often lead families to deprioritize daughters’ education—not due to lack of ability, but because of entrenched gender norms, with variations across regions. Yet change is possible. Educated Ghanaian women are already breaking these cycles—pursuing education, influencing policy, and investing in the next generation. The ripple effects are clear—healthier families, stronger economies, and more equitable societies. As Ghana advances toward the Sustainable Development Goals, this research underscores a vital truth: meaningful progress depends on transforming the gender norm beliefs that shape choices at home. Classrooms must become spaces of gender equity, and future research should explore how shifting norms can reshape parental attitudes over time. When families value daughters equally, the nation stands to gain immeasurably.

## Data Availability

No new data were created or analyzed in this study. Data sharing is not applicable to this article.
